# *In Vitro* Evaluation of Mangostanin as an Antimicrobial and Biocompatible Topical Antiseptic for Skin and Oral Tissues

**DOI:** 10.1021/acsptsci.4c00082

**Published:** 2024-04-19

**Authors:** Marta Munar-Bestard, Ana Rodríguez-Fernández, Joana Maria Ramis, Marta Monjo

**Affiliations:** † Cell Therapy and Tissue Engineering Group, Department of Fundamental Biology and Health Sciences, Research Institute on Health Sciences (IUNICS), University of the Balearic Islands, Ctra Valldemossa km 7.5, 07122 Palma, Spain; ‡ Health Research Institute of the Balearic Islands (IdISBa), 07120 Palma, Spain; § Department of Fundamental Biology and Health Sciences, University of the Balearic Islands, Ctra Valldemossa km 7.5, 07122 Palma, Spain

**Keywords:** mangostanin, 9-hydroxycalabaxanthone, hydrogel, antiseptic, chlorhexidine, skin infections, oral tissue infections

## Abstract

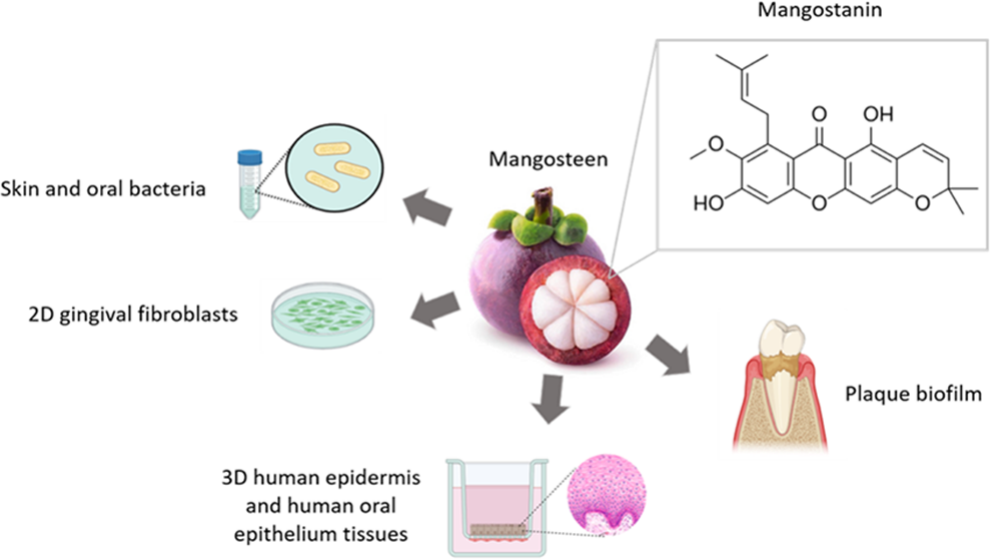

Skin and oral tissue infections pose significant health challenges worldwide, necessitating the exploration of new antiseptic agents that are both effective and biocompatible. This study evaluated the antibacterial efficacy and biocompatibility of mangostanin (MGTN), a xanthone derived from *Garcinia mangostana* L., against commercial antiseptics across various bacterial strains (*Porphyromonas gingivalis*, *Streptococcus mutans*, *Staphylococcus aureus*, *Staphylococcus epidermidis*, *Streptococcus pyogenes*, and *Cutibacterium acnes*) and *in vitro* models of skin and oral tissues. MGTN demonstrated significant antimicrobial activity against all tested pathogens concurrently exhibiting negligible cytotoxic effects on human gingival fibroblasts as well as on three-dimensional (3D) models of human epidermis and oral epithelium. Furthermore, using pooled human saliva, MGTN effectively inhibited plaque biofilm formation, suggesting its potential as a natural, biocompatible antiseptic for skin and oral health applications. These findings position MGTN as a promising candidate for further development into antiseptic formulations, offering a natural alternative to current synthetic options.

Antiseptics effectively treat living tissues by either killing or inhibiting microorganisms, thereby helping to prevent or reduce the risk of infection.^1,2^ However, while an antiseptic formulation may possess potent antimicrobial effects, it should not cause side effects such as irritation or toxicity when applied.^1,2^ Strikingly, recent evidence supports the presence of toxicity associated with antiseptics frequently utilized in clinical practice.^1^ Antiseptic products are used to prevent and treat local infections, such as periodontal diseases, skin infections, or chronic wound bacteria colonization.^2^ The most used antiseptics are chlorhexidine (CHX), povidone-iodine, hexetidine, eosin disodium, hydrogen peroxide, and undecylenamidopropyl betaine/polyhexanide.^1^ However, these antiseptics have some disadvantages, such as skin irritation, allergic reactions, reduction of fibroblast proliferation, migration, and viability.^1,3−6^ Moreover, the emergence of bacterial resistance poses a significant challenge to the long-term efficacy of these antiseptics. Bacteria can develop resistance mechanisms against antiseptics through various pathways, including cellular impermeability, biofilm formation, efflux and mutations at the target site, or overexpression of the target, rendering them less susceptible to antimicrobial agents over time.^7−9^

For oral diseases, the gelling substance hyaluronic acid (HA) and chitosan are often combined with antimicrobial substances such as CHX and enoxolone or with regeneration factors such as asiaticoside to promote tissue regeneration.^10^ However, these formulations have disadvantages, such as being toxic to cells.^10^ Especially, it has been reported that when CHX is used for a long time, it can cause ulcers on the oral mucosa, stain teeth and other oral surfaces, alter the taste, induce tissue cytotoxicity, and elevate levels of the potentially carcinogenic compound *p*-chloroaniline in saliva.^11,12^ Therefore, while antiseptics remain crucial in clinical practice, the prevalence of adverse effects and the emergence of bacterial resistance underscore the pressing need for the development of safer and more effective alternatives.

Thus, we focused on finding alternative antiseptics that were biocompatible while retaining excellent antimicrobial properties. Plants are valuable sources of novel bioactive compounds, which produce a wide variety of secondary metabolites that are effective against pathogens.^13^ Mangosteen pericarp and its bioactive xanthones have been reported to exhibit numerous biological activities, such as the inhibition of the growth of various types of bacteria and fungi, reduction of inflammation, and protection against oxidative stress, among other properties.^14−23^ Out of these xanthones, mangostanin (MGTN), also known as 9-hydroxycalabaxanthone, has demonstrated cytotoxicity against cancer cells, antioxidant and antimalarial properties, and antimicrobial activity against *Staphylococcus aureus*, *Bacillus cereus*, *Bacillus subtilis*, *Vibrio rotiferianus*, and *Vibrio campbellii.*(15,24−29)

Thus, in order to find a viable alternative for the treatment of skin and oral infections, we previously developed a chemically modified HA gel containing MGTN and showed its stability, biocompatibility, and antimicrobial effects against *Porphyromonas gingivalisin vitro* (unpublished results). MGTN was combined with a HA hydrogel, given its biocompatibility, nonimmunogenicity, and antimicrobial, anti-inflammatory, and tissue regeneration effects.^30−33^

In the present study, we aimed to further evaluate the antimicrobial activity of MGTN on selected skin and oral bacteria and to prove its biocompatibility on both two-dimensional (2D) gingival fibroblasts and three-dimensional (3D) human epidermis and 3D human oral epithelium tissues. In addition, we also evaluated its ability to prevent or reduce plaque biofilm accumulation in pooled human saliva. Four different studies were planned for this assessment using MGTN either in solution or formulated in a hyaluronic acid (HA) hydrogel.

## 1 Results

### 1.1 Antimicrobial Activity of MGTN in Solution on Different Representative Skin and Oral Bacteria

**Figure 1:**
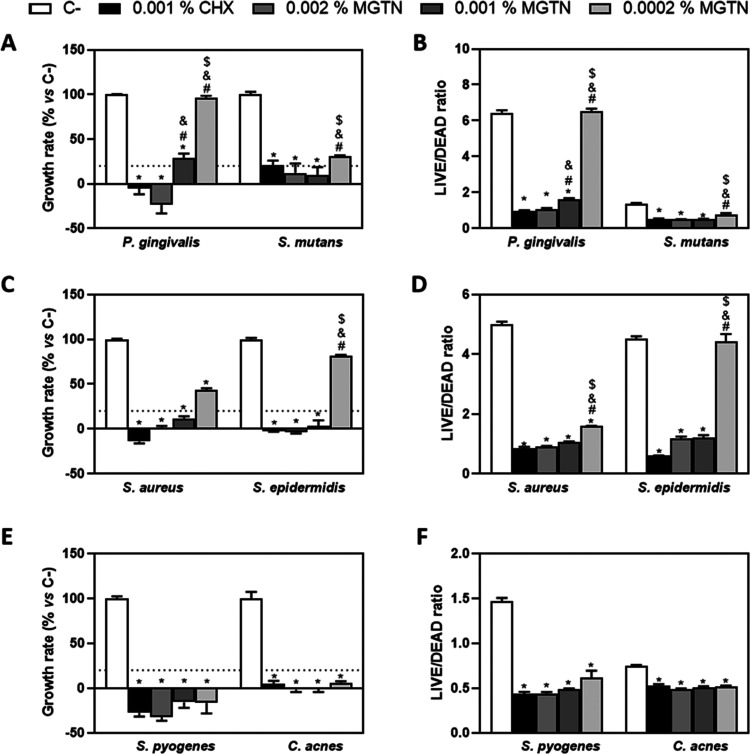
Antimicrobial activity of different concentrations (0.002, 0.001, and 0.0002%) of MGTN on *P. gingivalis* (for 10 h), *S. mutans* (for 5 h), *S. aureus* (for 3 h), *S. epidermidis* (for 10 h), *S. pyogenes* (for 24 h), and C. acnes (for 24 h). (A, C, E) *P. gingivalis*, *S. mutans*, *S. aureus*, *S. epidermidis*, *S. pyogenes*, and *C. acnes* growth rates cultured with different concentrations of MGTN. (B, D, F) *P. gingivalis*, *S. mutans*, *S. aureus*, *S. epidermidis*, *S. pyogenes*, and *C. acnes* LIVE/DEAD ratios cultured with different concentrations of MGTN. Data represent the mean ± SEM (*n* = 3). The negative control (C−) was the bacterial suspension without any treatment and the positive control (C+) was the bacterial suspension with CHX at 0.001%. Results were statistically compared by ANOVA and Bonferroni as post hoc for *P. gingivalis* LIVE/DEAD ratio, *S. mutans* growth rate, *S. mutans* LIVE/DEAD ratio, *S. aureus* LIVE/DEAD ratio, *S. epidermidis* growth rate, *S. pyogenes* growth rate, *S. pyogenes* LIVE/DEAD ratio, *C. acnes* growth rate, and *C. acnes* LIVE/DEAD ratio and by Kruskal–Wallis for *P. gingivalis* growth rate, *S. aureus* growth rate, and *S. epidermidis* LIVE/DEAD ratio: *p < 0.05 treatment *vs* C–. ^#^*p* < 0.05 treatment *vs* 0.001% CHX. ^&^*p* < 0.05 treatment *vs* 0.002% MGTN. ^$^*P* < 0.05 treatment *vs* 0.001% MGTN.

In this study, we evaluated the antibacterial properties of MGTN in solution against diverse types of bacteria commonly found in the oral cavity and on the skin. Figure 1 shows that a concentration of 0.002% MGTN incubated on *P. gingivalis*, a concentration of 0.001% MGTN incubated on *Streptococcus mutans*, *S. aureus*, and *Staphylococcus epidermidis*, and a concentration of 0.0002% MGTN incubated on *Streptococcus pyogenes* and *Cutibacterium acnes* were sufficient to eliminate more than 80% of the bacteria and presented a low LIVE/DEAD ratio of bacteria, which is similar to the efficacy of the most common antiseptic used for the skin, such as CHX at 0.001%.

Moreover, a concentration of 0.001% MGTN incubated on *P. gingivalis* and a concentration of 0.002% of MGTN incubated on *S. mutans*, *S. aureus*, and *S. epidermidis* produced a significantly high inhibition of the bacterial growth rate and a lower LIVE/DEAD ratio with respect to the negative control, which indicates that they decreased bacterial survival.

### 1.2 Inhibition of Bacterial Plaque Development from Human Saliva

**Figure 2:**
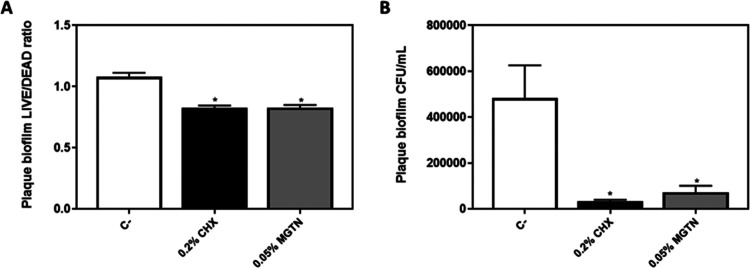
Effect of 0.05% MGTN and 0.2% CHX on the (A) LIVE/DEAD ratio and (B) CFU/mL of the bacterial plaque biofilm. Data represent the mean ± SEM (*n* = 6). Results were statistically compared by ANOVA and Bonferroni as post hoc for LIVE/DEAD and Kruskal–Wallis for CFU/mL: *p < 0.05 treatment *vs* C–.

We created a biofilm on glass slides using saliva from different healthy donors and tested the comparison of MGTN and CHX effects on plaque biofilm accumulation (Figure 2). The results showed that the treatment with 0.05% MGTN reduced plaque biofilm bacteria development with results equal to 0.2% CHX, the positive control, having a significant bactericidal effect (Figure 2A) and also an important reduction in the amount of colony-forming units (CFUs) per milliliter of the isolated plaque biofilm (Figure 2B).

### 1.3 Biocompatibility of MGTN on the 2D Cell Culture of Gingival Fibroblasts

**Figure 3:**
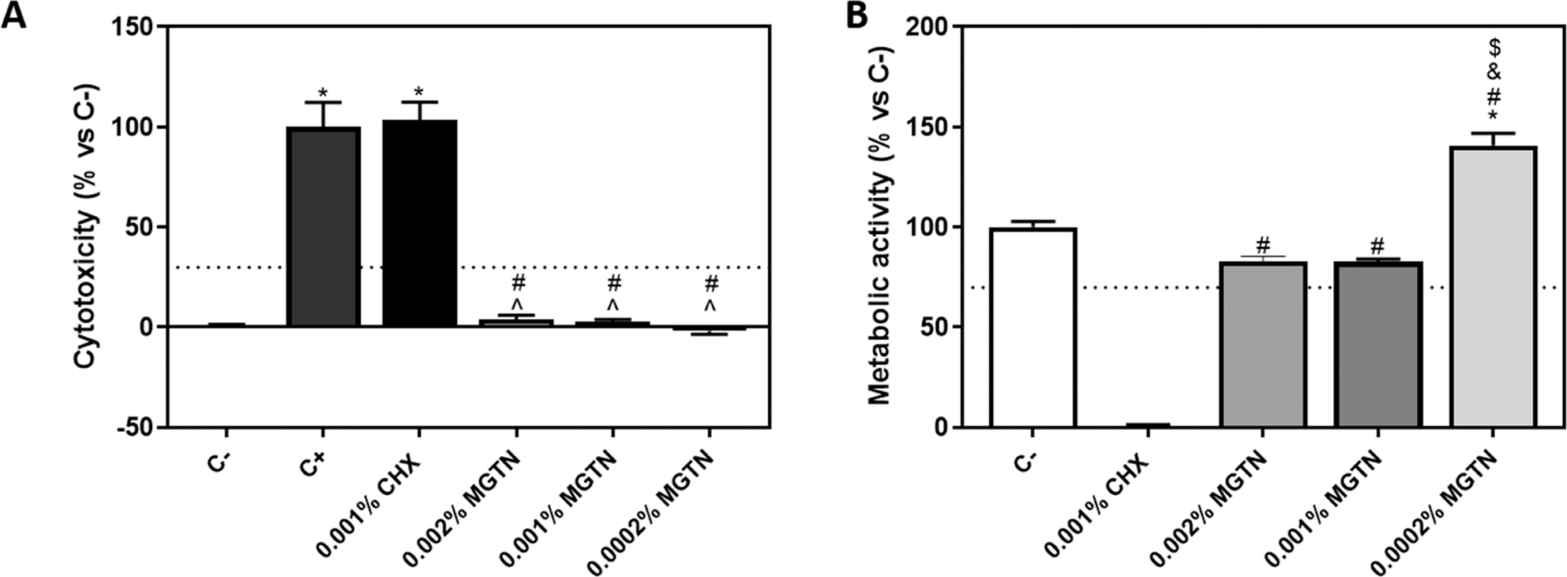
*In vitro* study 2D cell culture model of different concentrations of MGTN. (A) LDH activity, an indicator of cytotoxicity, was measured in culture media after the application of different concentrations of MGTN for 48 h. The negative control (C−) (0% toxicity) was obtained from cells without treatment. The positive control (100% toxicity) was obtained from cells seeded on plastic and treated with 1% triton X-100. (B) Metabolic activity, an indicator of the viability of cells, was measured in cultured media after the application of different concentrations of MGTN for 48 h. The negative control (100% viability of cells) was obtained from cells without treatment. Values represent the mean ± SEM (*n* = 6). Results were statistically compared by ANOVA and Bonferroni as post hoc: *p < 0.05 treatment *vs* C–. ^∧^*p* < 0.05 treatment *vs* C+. ^#^*p* < 0.05 treatment *vs* 0.001% CHX. ^&^*p* < 0.05 treatment *vs* 0.002% MGTN. ^$^*p* < 0.05 treatment *vs* 0.001% MGTN.

We used the lactate dehydrogenase (LDH) assay as an indicator of cytotoxicity, as this enzyme leaks out through the plasma membrane of damaged cells. As shown in Figure 3A, 0.001% CHX produced a release of LDH similar to that of the positive control, which represents 100% cytotoxicity. In contrast, 0.002, 0.001, and 0.0002% MGTN showed good biocompatibility on gingival fibroblasts, showing very similar values to the negative control. The metabolic activity was also used as an indicator of cell viability. As shown in Figure 3B, 0.001% CHX produced a significantly lower metabolic activity with respect to the negative control and all treatments. In contrast, 0.002 and 0.001% MGTN showed good results of biocompatibility on gingival fibroblasts, which were very similar to the negative control. However, 0.0002% MGTN showed better results of biocompatibility on gingival fibroblasts than the negative control and 0.002 and 0.001% MGTN.

### 1.4 Biocompatibility of Antiseptic Skin and Periodontal Gels on 3D Tissues and Antimicrobial Activity

#### 1.4.1 Antiseptic Skin Gels

**Figure 4:**
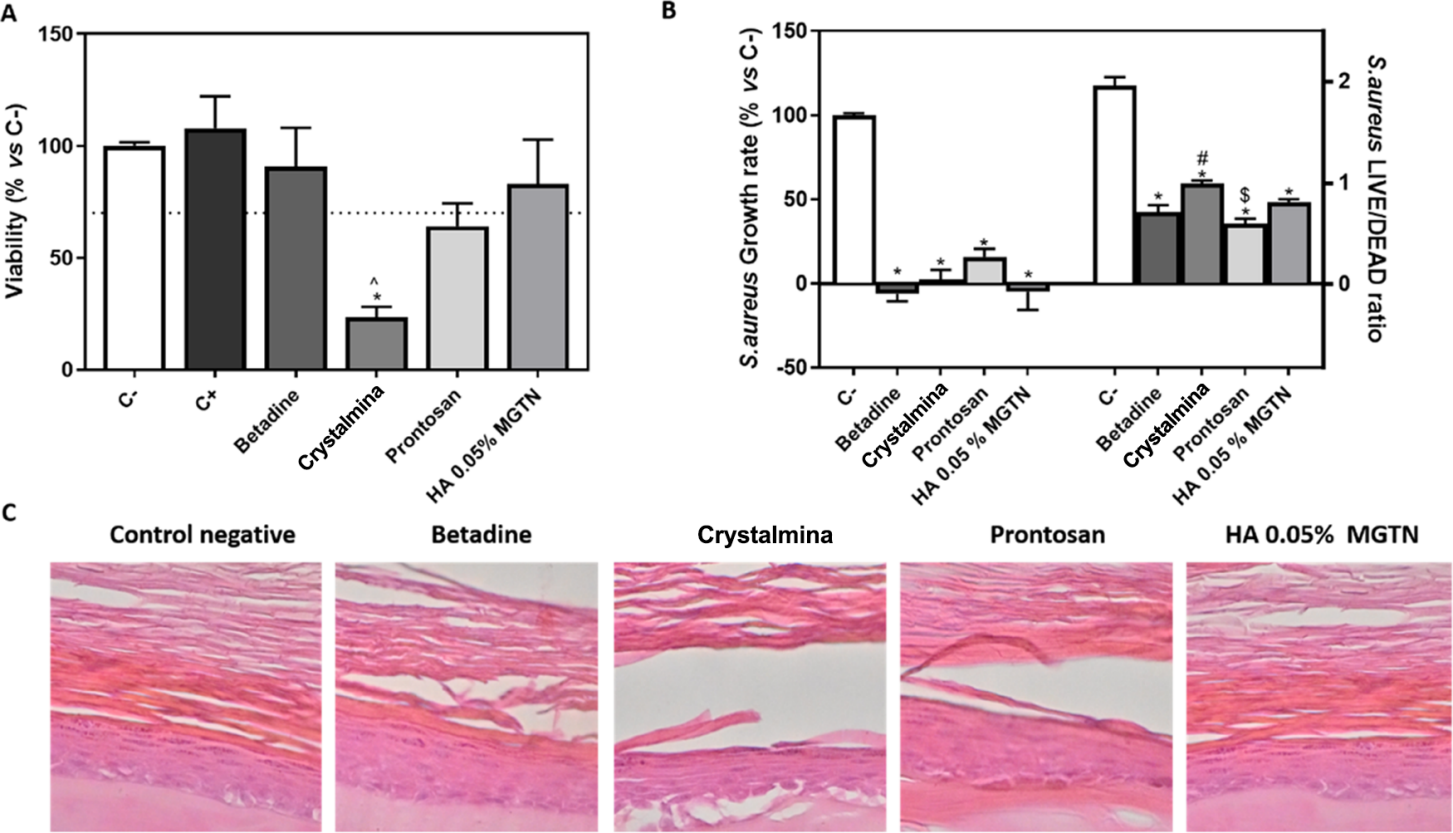
Biocompatibility and antimicrobial test of antiseptic skin gels. (A) MTT viability assay in RHE tissue after the application of antiseptic skin gels. Results are expressed as % *vs* C– that was set to 100%. C+ represents 5% SDS. (B) *S. aureus* growth rate and LIVE/DEAD ratio cultured for 3 h with different antiseptic skin gels. (C) Histological characterization of RHE. Data represent the mean ± SEM (*n* = 3). Results were statistically compared by ANOVA and Bonferroni as post hoc for MMT assay and for the *S. aureus* growth rate and by Kruskal–Wallis for *S. aureus:* *p < 0.05 treatment *vs* C–. ^∧^*p* < 0.05 treatment *vs* C+. ^#^*p* < 0.05 treatment *vs* Betadine gel. ^$^*p* < 0.05 treatment *vs* Crystalmina gel.

We used the MTT assay to evaluate the viability of reconstructed human epidermis (RHE) tissue after 3 h of treatment with antiseptic skin gels by measuring the MTT reduction by mitochondrial reductase enzymes. The results shown in Figure 4A indicate that the modified HA gel with 0.05% MGTN, Betadine gel, and Prontosan gel was biocompatible on EpiSkin tissue, similar to untreated epidermis control tissue (C−). However, the commercial Crystalmina gel containing 1% CHX had a toxic effect on the tissue, with only 27% of viability. In contrast, the 5% SDS positive control (C+) in this study did not cause tissue death.

Moreover, Figure 4B shows that all antiseptic skin gels exhibited high antibacterial activity against *S. aureus*, a typical bacterium associated with skin infections.

We performed histology to confirm the morphology and viability of the RHE tissues after the applications of the different antiseptic skin gels for 3 h, as shown in Figure 4C. Images for Betadine, Prontosan, and HA 0.05% MGTN confirm the good structure of human keratinocytes cultured in a collagen matrix, similar to the untreated control tissue. However, the images for Crystalmina gel containing 1% CHX show a more disrupted layer of keratinocytes on the tissue surface compared to the other treatments.

Moreover, in this study, we evaluated the impact of various antiseptic skin gel treatments on RHE tissue by measuring the release of the proinflammatory cytokine interleukin-1α (IL-1α) after application of the treatments. None of the antiseptic skin gels and the negative control released detectable levels of IL-1α after 3 h of treatment (data not shown).

#### 1.4.2 Antiseptic Periodontal Gels

**Figure 5:**
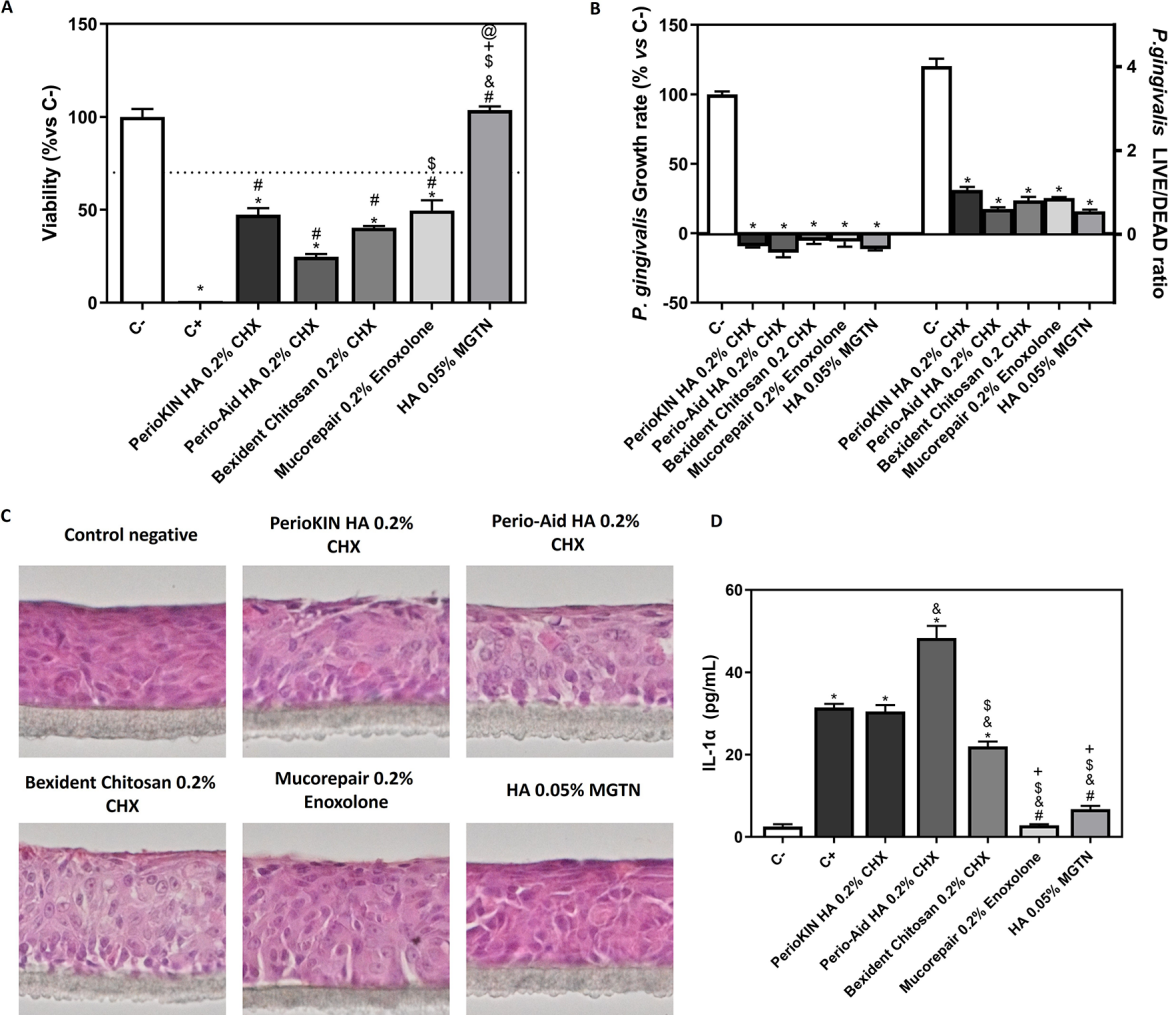
Biocompatibility and antimicrobial test of periodontal gels. (A) MTT assay in HOE tissue after the application of periodontal gels for 3 h. Results are expressed as % *vs* C- that was set to 100%. C+ represents 5% SDS. (B) *P. gingivalis* growth rate cultured at 10 h with different periodontal gels and *P. gingivalis* LIVE/DEAD ratio cultured for 10 h with different periodontal gels. (C) Histological characterization of HOE. Representative images were present for each treatment—detail of hematoxylin and eosin (H&E) staining of HOE at 400×. Total tissue disintegration was observed in C+ after the treatment with SDS 5% and they were not further processed for histological analysis. Values represent the mean ± SEM (*n* = 3). (D) Effect of different periodontal gels on IL-1α release by HOE after 3 h of treatment. Results were statistically compared by ANOVA and Bonferroni as post hoc for MMT assay and by Kruskal–Wallis for *P. gingivalis* growth rate, *P. gingivalis* LIVE/DEAD ratio, and IL-1α levels: *p < 0.05 treatment *vs* C–. ^#^*p* < 0.05 treatment *vs* C+. ^&^*p* < 0.05 treatment *vs* Periokin HA 0.2% CHX. ^$^*p* < 0.05 treatment *vs* Perio-Aid HA 0.2% CHX. ^+^*p* < 0.05 treatment *vs* Bexident Chitosan 0.2% CHX. ^@^*pp* < 0.05 treatment *vs* Mucorepair 0.2% Enoxolone.

In addition, we evaluated the effects of different periodontal gels on human oral epithelium (HOE) tissue after 3 h of treatment. The results shown in Figure 5A indicate that the modified HA gel with 0.05% MGTN presented very good biocompatibility in HOE tissues, similar to that of untreated control tissues. However, the commercial gels containing 0.2% CHX with unmodified HA or chitosan (Periokin HA 0.2% CHX, Perio-Aid HA 0.2% CHX, and Bexident Chitosan 0.2% CHX) and the commercial gel containing 0.2% enoxolone (Mucorepair 0.2% Enoxolone) presented toxic effects on HOE, displaying a viability below 70%.

In addition, all periodontal gels had high antibacterial activity against *P. gingivalis* (Figure 5B), a characteristic bacterium of periodontal diseases.

We performed histology to confirm the morphology and viability of the HOE tissues after the different treatments, as shown in Figure 5C. The images for the negative control, Perio-Aid HA 0.2% CHX, Bexident Chitosan 0.2% CHX, Mucorepair 0.2% Enoxolone, and HA 0.05% MGTN confirmed the good structure of the mucosa of the oral cavity. However, images for Periokin HA 0.2% CHX showed a destroyed layer of cells on the tissue surface compared with the other treatments.

Furthermore, we evaluated the release of the proinflammatory cytokine IL-1α after 3 h of treatment. The commercial gels containing 0.2% CHX with HA or chitosan (Periokin HA 0.2% CHX, Perio-Aid HA 0.2% CHX, and Bexident Chitosan 0.2% CHX) presented significantly higher levels of IL-1α compared to the negative control (C−). In contrast, the commercial gel containing 0.2% enoxolone (Mucorepair 0.2% Enoxolone) and HA 0.05% MGTN presented significantly lower levels of IL-1α compared to the positive control (C+) (Figure 5D).

## 2 Discussion

The present work demonstrates the antimicrobial efficacy of MGTN against the main skin and oral bacteria and its excellent biocompatibility, outperforming the gold standard antiseptic CHX and other marketed products.

Our results show the antimicrobial effect of MGTN against all of the bacteria evaluated, either the Gram-positive (*S. aureus*, *S. epidermidis*, *S. mutans*, *S. pyogenes*, and *C. acnes*) or the Gram-negative anaerobic bacteria *P. gingivalis*. Thus, it is a good candidate as an antiseptic for preventing periodontal and skin diseases. We have selected *P. gingivalis* as the main key pathogen in periodontal disease^34^ and *S. aureus* for being the main cause of nosocomial bacteremia in different parts of the world due to the factors of high virulence and pathogenicity.^35^ In addition, *S. epidermidis*, which resides on human skin and mucosal surfaces, can lead to various infections and form durable biofilms on surfaces, especially on polymeric plastic or metal.^36^*S. pyogenes* is one of the most significant bacterial pathogens affecting human skin, causing impetigo, cellulitis, and severe conditions such as sepsis and necrotizing fasciitis, leading to substantial morbidity/mortality and a notable public health impact.^37^ Last, *C. acnes* is a commensal bacterium of the skin that inhabits follicles and glands and is also found in mucosal regions, exhibiting pathogenicity in infections spanning cutaneous, digestive, cardiovascular, and orthopedic contexts, particularly in relation to implants.^38^ Our results are in agreement with previous studies reporting the antibacterial efficacy of various xanthones,^39−43^ including MGTN^25,26,44^ or mangosteen extracts.^45,46^

In addition, we also showed the efficacy of 0.05% MGTN to prevent bacterial plaque biofilm formation from saliva, comparable to the effectiveness of 0.2% CHX. Dental plaque is a biofilm of oral microorganisms that accumulates on nonshedding tooth surfaces, contributing to periodontal disease and dental caries. It comprises around 700 bacterial species, with *S. mutans* being a key cariogenic species.^47^ These results are in line with other reported research showing that xanthones can damage the mechanical stability of the *S. mutans* biofilm and inhibit glucosyltransferase enzyme activity, which results in the inhibition of biofilm growth.^47^ Mangosteen pericarp extract has demonstrated promise as a plaque inhibitor in mouthwashes,^48,49^ and a soluble film of xanthone α-mangostin exhibited antimicrobial activity against oral pathogens and inhibited biofilm formation *in vitro*.^13,42^

Once the effectiveness of MGTN as an antiseptic either in solution or in formulation was confirmed, we also evaluated its safety through biocompatibility assessment, confirming the lack of cytotoxicity on the 2D culture *in vitro* model of human gingival fibroblasts by the low LDH activity levels and improved metabolic activity induced by MGTN treatment. To go further, commercial 3D models of human epidermis and oral mucosa epithelium were used to test different gels. In both 3D models, we found that modified HA combined with 0.05% MGTN did not cause any deleterious effect. These results are consistent with our previous study, which demonstrated the lack of the cytotoxicity effect of modified HA combined with MGTN on the 3D model of the gingival tissue equivalent (Munar-Bestard et al.; Mangostanin hyaluronic acid hydrogel as an effective biocompatible alternative to chlorhexidine, 2023; manuscript submitted for publication). In addition, in the commercial 3D model of oral mucosa epithelium, the treatment with HA 0.05% MGTN gel showed low IL-1α levels, contrary to the positive control or to the gels containing CHX, thus showing excellent biocompatibility with tissues, as observed for the Betadine and Prontosan gels, whereas Crystalmina gel that contains 1% CHX exhibited a higher cytotoxicity, in concordance with the literature.^50−52^ The lack of effect in cell viability induced by the positive control on the 3D model of human epidermis can be explained by the keratinized external layer of this tissue and/or to the relatively low SDS concentration used in this assay, evidencing the low sensibility of the 3D model of human epidermis tissue for MTT.^53^ In contrast, comparing the effects of HA with 0.05% MGTN with the commercial periodontal gels, we observed that the commercial periodontal gels containing 0.2% CHX exhibited a higher cytotoxicity in the 3D oral mucosa model, in line with CHX effects.^10^ Treatment with gels containing 0.2% CHX also resulted in significantly higher levels of IL-1α, a proinflammatory cytokine that has a specific role in inflammation, immunity, tissue breakdown, and tissue homeostasis,^54,55^ directly related to the progression of periodontal diseases,^54,56^ and a sensitive indicator of acute irritation in reconstructed human epidermis.^57^ Additionally, the Mucorepair 0.2% Enoxolone gel that contained 0.2% enoxolone and 0.3% asiaticoside exhibited a higher cytotoxicity in the commercial 3D model of the oral mucosa. Asiaticoside has been shown to have an antiproliferative and toxic effect on cells, which explains the results obtained in the present study, with a demonstrated antibacterial action and high cytotoxicity on the oral mucosa.^58,59^ Furthermore, histological images revealed a damaged layer of cells on the tissue surface-treated with Periokin HA 0.2% CHX compared to other treatments containing 0.2% CHX, such as Perio-Aid HA 0.2% CHX. This discrepancy might be attributed to the presence of panthenol in the gel, which offers regenerative and anti-inflammatory properties while maintaining skin softness and elasticity.^60^

The differential mechanisms of action between CHX and xanthones may account for the observed disparities in antimicrobial activity and cytotoxicity. CHX, a synthetic antiseptic, primarily targets bacterial cell membranes. Its positively charged molecules interact with the negatively charged bacterial cell membrane, leading to increased permeability. This disruption causes the leakage of essential cellular components, ions, and interfaces with DNA and protein functions, culminating in bacterial cell death.^9,61^ In contrast, xanthones are thought to inhibit specific bacterial enzymes, impede bacterial DNA replication, and interfere with biofilm formation, offering a more multifaceted approach to combat bacteria.^45,62^ When considering their impact on bacteria, xanthones, including MGTN, demonstrate a multifaceted mechanism of action. The isoprenyl groups within the xanthone structure facilitate interactions with bacterial lipid alkyl chains, allowing for penetration into bacterial cells. Once inside, they disrupt the integrity of the cytoplasmic membrane and damage intracellular components, compromising bacterial viability.^41,63^ Furthermore, the carbonyl groups present in the xanthone structure enable targeting of protein cell membranes, enzymes, and the structural integrity of biofilm matrix polymers.^64^ This comprehensive approach renders xanthones with potent antibacterial properties, capable of effectively combating bacterial infections. While both CHX and xanthones target bacterial cells, CHX primarily focuses on membrane disruption and binding to cellular components, whereas the mechanism of xanthones seems to involve enzyme inhibition. A study even demonstrated the potential of xanthone derivatives as antibiotics by inhibiting enzyme I of the bacterial phosphoenolpyruvate-dependent phosphotransferase system, a feature ubiquitous in eubacteria but absent in eukaryotes.^65^ This might explain why xanthones can inhibit bacterial growth without causing cytotoxicity in human tissues.

Moving forward, our next objective is to validate the efficacy of the modified HA gel incorporating MGTN in an *in vivo* inflammatory animal model of periodontitis. This step is crucial to confirm not only the gel’s biocompatibility but also its effectiveness in combating bacterial infection, reducing inflammation, and promoting tissue regeneration. Additionally, we intend to assess the long-term effects and safety profile of the gel to establish its potential for clinical application. Furthermore, we will explore the possibility of combining the MGTN-containing gel with other therapeutic agents or treatment modalities to enhance the efficacy and versatility in managing periodontal diseases. Ultimately, the successful validation of this innovative approach holds promising prospects for revolutionizing the treatment landscape of periodontitis and other related inflammatory conditions.

In conclusion, the natural bioactive compound MGTN, formulated either in solution or in the hydrogel form, has demonstrated excellent biocompatible and antimicrobial properties in several *in vitro* models. Therefore, a new treatment could be developed in the field of cosmetics for the treatment of skin and oral diseases as an alternative to products currently on the market, which show excellent antimicrobial activity but lack biocompatibility. Importantly, the findings underscore the significance of exploring the potential of MGTN in the development of new antiseptic formulations with broader applications in clinical settings. Such advancements hold promise for improving therapeutic outcomes and enhancing patient care in diverse medical contexts.

## 3 Methods

### 3.1 Mangostanin

Mangostanin (MGTN), chemically known as 5,9-dihydroxy-8-methoxy-2,2-dimethyl-7-(3-methylbut-2-enyl)pyrano[3,2-*b*]xanthen-6-one, was sourced from Cymit Qumica S.L., Barcelona, Spain. We stocked it in 2% dimethyl sulfoxide (DMSO) aliquots at −70 °C and diluted it to obtain the desired concentration and eliminate the DMSO toxicity risk.

### 3.2 Formulations Used in the Study

**Figure 6:**
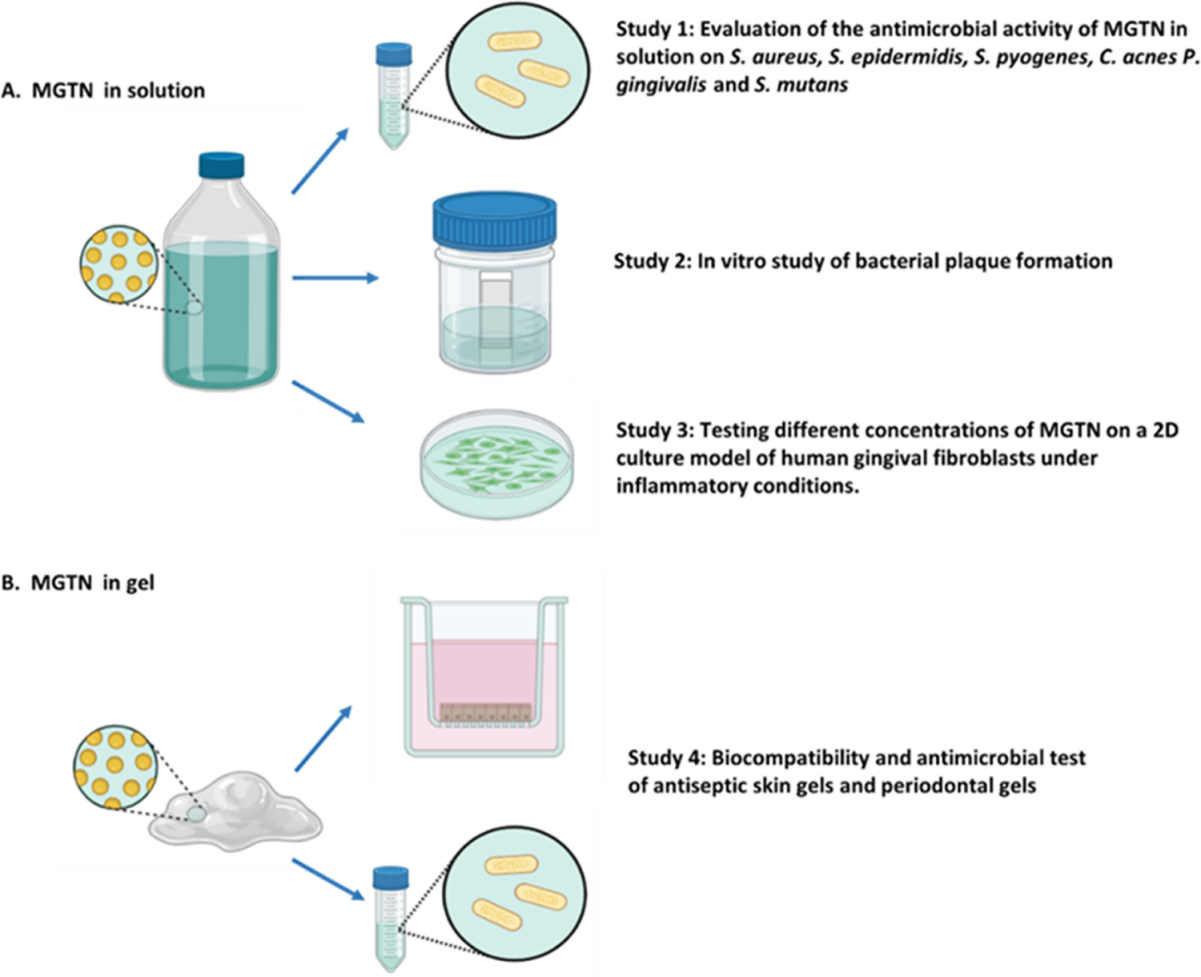
Representative diagram of the different studies carried out in this research. (A) Studies in which MGTN is evaluated in solution. (B) Studies in which MGTN is formulated in a modified hyaluronic gel.

In this work, we used MGTN in solution or formulated it in a modified hyaluronic gel to carry out different studies (Figure 6). On one hand, we employed different concentrations of MGTN in solution to evaluate its antimicrobial activity against various skin bacteria (*S. aureus*, *S. epidermidis*, *S. pyogenes*, and *C. acnes*) or oral bacteria (*P. gingivalis* and *S. mutans*) (**Study 1**). We also evaluated the prevention or reduction of plaque biofilm development from the saliva of healthy donors *in vitro* using MGTN in solution (**Study 2**) and assessed its biocompatibility on conventional 2D cultures of gingival fibroblasts (**Study 3**). On the study, we employed four different commercial skin antiseptic gels and a modified HA gel with 0.05% MGTN to determine the biocompatibility on the 3D cell culture of reconstructed human epidermis and the antibacterial activity against *S. aureus*. Similarly, in the study, we employed four different commercial periodontal gels and a modified HA gel with 0.05% MGTN to evaluate the biocompatibility on the 3D cell culture of human oral epithelium and the antibacterial activity against *P. gingivalis* (**Study 4**).

### 3.3 Study 1: Evaluation of the Antimicrobial Activity of MGTN in Solution on *S. aureus*, *S. epidermidis*, *S. pyogenes*, *C. acnes*, *P. gingivalis*, and *S. mutans*

#### 3.3.1 Culture and Proliferation Assay of Different Bacteria

*S. epidermidis* (4814, obtained from the Spanish Type Culture Collection (CECT), University of Valencia, Spain) and *S. aureus* (ATCC 29213, Manassas, VA) were grown from frozen stocks in Luria–Bertani (LB) broth (Scharlab, Barcelona, Spain); *S. pyogenes* (Son Espases University Hospital (HUSE), Department of Microbiology, Spain) was grown from frozen stocks in tryptic soy broth (TSB) (Scharlab); and *S. mutans* (479, CECT) was grown from frozen stocks in brain heart infusion (BHI) broth, all at 37 °C under aerobic conditions in an orbital shaker (180 rpm) for 24–48 h.

*C. acnes* (5684 CECT, Valencia, Spain) was grown on TSB (Scharlab), supplemented with 0.5 g/L l-cysteine hydrochloride (Thermo Fisher Scientific, Waltham, MA), 1 mg/L hemin (Thermo Fisher Scientific), and 5 mg/L glucose (Scharlab), and *P. gingivalis* (ATCC 33277TM) was grown on BHI broth (Scharlab), supplemented with 0.5 g/L l-cysteine hydrochloride (Thermo Fisher Scientific), 5 mg/L hemin (Thermo Fisher Scientific), and 1 mg/L vitamin K (Thermo Fisher Scientific), all at 37 °C under anaerobic conditions (10% H_2_, 10% CO_2_, and 80% N_2_) achieved with an Oxoid AnaeroGen sachet (Thermo Fisher Scientific) for 24–72 h.

After overnight incubation, we incubated 1 mL of the different bacterial suspensions with increasing concentrations of MGTN (0.002, 0.001, and 0.0002%) for different times depending on the bacteria to select exponential growth: 3 h (*S. aureus*), 5 h (*S. mutans*), 10 h (*S. epidermidis* and *P. gingivalis*), and 24 h (*S. pyogenes* and *C. acnes*). We used the bacterial suspension without treatment as a negative control (C−), and CHX at 0.001% served as a positive control for bacterial growth inhibition. In preliminary studies, we tested different concentrations of CHX and found that the optimal concentration for the positive control was 0.001%. We measured the optical density (OD) at 600 nm with a plate spectrophotometer (PowerWave Ht, Biotek instruments, Winooski, VT) at 0 and 3, 5, 10, or 24 h depending on the bacteria to determine bacterial proliferation. We calculated the bacterial growth rate (μ) during the exponential growth phase following the equation ln OD*_t_* – ln OD_0_ = μ × (*t* – *t*0). We determined the LIVE/DEAD ratio of the different types of bacteria using the LIVE/DEAD BacLight bacterial viability kit (Invitrogen, Thermo Fisher Scientific), following the manufacturer′s instructions. We conducted the assays in triplicate (*n* = 3).

### 3.4 Study 2: *In Vitro* Study of Bacterial Plaque Development

We conducted an *in vitro* study using pooled human saliva as a test substrate to compare the ability of MGTN *vs* CHX to prevent or reduce plaque biofilm development. We collected and pooled 150 mL of human saliva from 10 different donors. Then, we pipetted 15 mL of the pooled saliva supplemented with 0.1% sucrose (Scharlab into sterile 40 mL containers (Deltalab, Barcelona, Spain)) and submerged a glass microscope slide (Thermo Fisher Scientific) into 2 cm of saliva in each container and incubated at 37 °C for bacterial biofilm formation. Twice a day, we immersed the slide in the TSB (Scharlab) culture medium for 2 min. On days 2 and 3, we treated the biofilm with different groups: (1) a solution containing 0.05% MGTN (*n* = 6), (2) a solution containing 0.2% CHX (*n* = 6), and a solution containing water (C−) (*n* = 6), by immersing the slides for 2 min in the test of the assigned product. On day 4, we dipped the biofilms 3 times in saline and placed them in 5 mL of sterile saline. Then, we gently scraped the surfaces of the slides with a sterile scraper to collect adherent bacteria. The removed biofilms were subjected to sonication (Sonifier 250; Branson Ultrasonics Corporation, CT) using three 15 s pulses with a power of 7 W. We used the homogenized bacterial suspension to determine the bacterial viability both by quantification of CFU/mL and by LIVE/DEAD assay.

For the bacterial viability assay, we serially diluted an aliquot (0.1 mL) of the homogenized bacterial suspension and plated it on TSB agar. We then incubated the plates in 5% CO_2_ at 37 °C for 48 h and determined the number of CFU/mL. Moreover, we used an aliquot (0.1 mL) of the homogenized bacterial suspension to determine the LIVE/DEAD ratio of bacteria using the LIVE/DEAD BacLight bacterial viability kit (Invitrogen), following the manufacturer′s instructions.

### 3.5 Study 3: Testing Different Concentrations of MGTN on a 2D Culture Model of Human Gingival Fibroblasts under Inflammatory Conditions

#### 3.5.1 Immortalized Human Gingival Fibroblasts (iHGFs)

Immortalized human gingival fibroblasts-hTERT (iHGFs) (Applied Biological Materials Inc., Richmond, BC, Canada) were grown at 37 °C in an atmosphere of 5% CO_2_ using a fibroblast medium as previously described.^24^ We seeded cells in 48-well plates at a density of 2 × 10^4^ cells/well. At confluence, we used these cells for the biocompatibility assays.

#### 3.5.2 iHGFs Treated with Different Concentrations of MGTN

We induced inflammation in confluent cells by lipopolysaccharides (LPSs) from *P. gingivalis* (Invivo Gen, San Diego, CA) at 1 μg/mL and then treated with different concentrations of MGTN (0.002, 0.001, and 0.0002%) in fibroblast medium with 50 μg/mL ascorbic acid (Sigma-Aldrich, St. Louis, MO) for 48 h. Fibroblast medium with 50 μg/mL ascorbic acid (Sigma-Aldrich) and without a biomolecule served as a negative control, and CHX (Abcam, Cambridge, U.K.) at 0.001% served as a positive control. In preliminary studies, we tested different concentrations of CHX and found that the optimal concentration for the positive control was 0.001%.

#### 3.5.3 Cell Cytotoxicity

To estimate the cytotoxicity of the different concentrations of MGTN, we used the presence of LDH in culture media 48 h after the proinflammatory stimulus with LPS and the treatment as an index of cell death, following the manufacturer’s instructions (cytotoxicity detection kit, Roche Diagnostics, Mannheim, Germany), as previously described. We conducted three independent experiments, with two replicates at each condition (*n* = 6).

#### 3.5.4 Metabolic Activity

We evaluated the total metabolic activity after 48 h of treatment for iHGFs using Presto Blue reagent (Life Technologies, Carlsbad, CA) following the manufacturer’s protocol. We set nontreated cells as 100%. We conducted three independent experiments, with two replicates at each condition (*n* = 6).

### 3.6 Study 4: Biocompatibility and Antimicrobial Test of Antiseptic Skin Gels and Periodontal Gels

In this study, we determined the biocompatibility of MGTN formulated with a modified HA hydrogel and compared it to that of commercial antiseptic skin and periodontal gels using different 3D tissue models. Additionally, we used both *S. aureus* and *P. gingivalis* as a representative of bacterial skin and periodontal infection to determine their antimicrobial activity.

#### 3.6.1 Different Antiseptic Gels

In the present study, we used three different commercial antiseptic skin gels (A–C), four commercial periodontal gels (D–G), and a modified HA gel with 0.05% 9-hydroxycalabaxanthone (MGTN)(H), specifically: (**A**) Betadine 10% topical gel (Mylan, West Virginia), containing povidone-iodine as an antiseptic, identified as Betadine; (**B**) Crystalmina film 1% topical gel (Laboratorios SALVAT, Barcelona, Spain), containing 1% of CHX as an antiseptic, identified as Crystalmina; (**C**) Prontosan gel (B. Braun, Melsungen, Germany), containing 0.1% undecylenamidopropyl betaine and 0.1% polyhexanide as antiseptics, identified as Prontosan; (**D**) PerioKIN hyaluronic 1% (Laboratorios Kin, Barcelona, Spain), containing 0.2% CHX and 1% HA, identified as PerioKIN HA 0.2% CHX; (**E**) Perio-Aid gel bioadhesive (Dentaid SL, Barcelona, Spain), containing 0.2% CHX, 0.2% HA, and 5% panthenol, identified as Perio-Aid HA 0.2% CHX; (**F**) Bexident post gel periodontal (ISDIN, Barcelona, Spain), containing 0.2% CHX and 0.5% chitosan, identified as Bexident Chitosan 0.2% CHX; (**G**) Lacer mucorepair gel (Lacer SA, Barcelona, Spain), containing 0.2% enoxolone, 0.2% HA, and 0.3% asiaticoside, identified as Mucorepair 0.2% Enoxolone; and (**H**) Modified HA hydrogel with 0.05% MGTN synthesized and characterized as previously described (unpublished results), identified as HA 0.05% MGTN.

#### 3.6.2 Reconstructed Human Epidermis and Human Oral Epithelium

We used EpiSkin small/reconstructed human epidermis (EpiSkin, Lyon, France) (RHE) as an *in vitro* model to perform the biocompatibility test. The RHE model is composed of normal human keratinocytes cultured on a collagen matrix at the air–liquid interface and is histologically similar to the *in vivo* human epidermis. This model exists at different stages of maturity; in our study, tissues were obtained at day 13.

We used the SkinEthic human oral epithelium (HOE) tissue model (EpiSkin, Lyon, France) as an *in vitro* model to perform the biocompatibility test. The HOE model is composed of TR146 cells (derived from a squamous cell carcinoma of the buccal mucosa) cultivated on an inert polycarbonate filter at the air–liquid interface in a chemically defined medium. This model forms an epithelial tissue devoid of stratum corneum resembling histologically the lining mucosa of the oral cavity.

The inserts containing RHE (size 0.38 cm^2^) and HOE (size 0.5 cm^2^) were shipped at room temperature in a multiwell plate filled with an agarose-nutrient solution in which they are embedded. Upon arrival, we processed the tissues following the manufacturer’s protocol. Briefly, we removed the RHE and HOE tissues from the agarose-nutrient solution and placed them in a plate previously filled with the EpiSkin maintenance medium (EpiSkin, Lyon, France) for RHE and SkinEthic maintenance medium (EpiSkin, Lyon, France) for HOE at room temperature. We then incubated the tissues in a cell incubator at 37 °C, 5% CO_2_, and saturated humidity for 48 h.

#### 3.6.3 RHE and HOE Treated with the Different Antiseptic Gels

After 48 h of incubation, we treated the RHE surface with the different antiseptic skin gels and the HA hydrogel with 0.05% MGTN or with the positive or negative control for 3 h. Similary, HOE was surface-treated with the different periodontal gels and the HA hydrogel with 0.05% MGTN or with a positive or negative control for 3 h. We assessed the viability of the RHE and HOE tissues using the MTT assay. For each time point, we used three tissue samples per group. We applied 40 μL of the different skin antiseptic gels on top of the reconstructed RHE samples and 50 μL of the different periodontal gels on top of the HOE samples and incubated at 37 °C for 3 h. For the negative control (C−), we used 1:1 Milli-Q water dilution in PBS and for the positive control (C+), we prepared a 10% SDS (Sigma-Aldrich, St. Louis, MO) solution in water and diluted 1:1 in PBS with a final concentration of 5% SDS, applying the same volume as the treatments. We conducted the assays in triplicate (*n* = 3). We used SDS at 5% concentration because it serves as a reliable positive control in tissue experiments, ensuring consistent disruption of cell membranes and validating experimental procedures.

#### 3.6.4 MTT

At the end of the incubation period with different gels, we washed the RHE and HOE tissues with PBS (Biowest) and incubated them with 400 μL of 0.5 mg/mL MTT (3-(4,5-dimethylthiazol-2-yl)-2,5-diphenyltetrazolium bromide (Thermo Fisher Scientific)). After 3 h of incubation at 37 °C and 5% CO_2_, we incubated the cultures with 3 mL of isopropanol (Sigma-Aldrich) for 24 h at RT. We measured the optical density at 570 nm (reference filter: 690 nm). Results are expressed as a percentage of viability compared with the negative control.

#### 3.6.5 Cytokine Levels of Interleukin-1α (IL-1α)

We performed the detection of IL-1α from cell culture media after 3 h of treatment with periodontal and antiseptic skin gels on HOE and RHE cultures using commercially available ELISA kits according to the manufacturer’s instructions (Thermo Fisher Scientific).

#### 3.6.6 Histology

We fixed the RHE and HOE tissues in 4% paraformaldehyde (PFA) (Scharlab) and processed for paraffin embedment. Subsequently, we cut paraffin sections (6 μm) and stained them with hematoxylin and eosin (H&E) for histological examination. We visualized the stained tissue sections using a BX60 microscope (Olympus, Tokyo, Japan) and captured images at 400× with a Nikon D5600 camera (Nikon, Tokyo, Japan) at a 1/1250 shutter speed and ISO 20,000.

#### 3.6.7 Antimicrobial Test of Antiseptic Skin Gels on *S. aureus*

We cultured and treated the bacteria *S. aureus* with different commercial antiseptic skin gels and the HA hydrogel with 0.05% MGTN. We diluted the gels in the bacterial media at a concentration of 5% (v/v), and we conducted the experiment following the procedure outlined in Section 3.3.1.

#### 3.6.8 Antimicrobial Test of Antiseptic Oral Gels on *P. gingivalis*

We cultured and treated the bacteria *P. gingivalis* with different commercial periodontal gels and HA formulated with 0.05% MGTN. We diluted the gels in the bacterial media at a concentration of 5% (v/v), and we conducted the experiment following the procedure outlined in Section 3.3.1.

### 3.7 Statistical Analyses

We presented all data as mean values ± SEM. We conducted the Shapiro–Wilk test to assume parametric or nonparametric distributions for the normality tests. Differences between groups were assessed by the Kruskal–Wallis test or by one-way ANOVA with Bonferroni as post hoc depending on their normal distribution. We utilized the SPSS program for Windows, version 17.0 (SPSS Inc., Chicago, IL) and GraphPad Prism (version 7, GraphPad Software Inc., La Jolla, CA). Results were considered statistically significant at *p*-values <0.05.
